# Effect of Central Sympathoinhibition With Moxonidine on Sympathetic Nervous Activity in Polycystic Ovary Syndrome—A Randomized Controlled Trial

**DOI:** 10.3389/fphys.2018.01486

**Published:** 2018-10-25

**Authors:** Soulmaz Shorakae, Elisabeth A. Lambert, Eveline Jona, Carolina Ika Sari, Barbora de Courten, John B. Dixon, Gavin W. Lambert, Helena J. Teede

**Affiliations:** ^1^Monash Centre for Health Research and Implementation, School of Public Health and Preventive Medicine, Monash University, Melbourne, VIC, Australia; ^2^Diabetes and Vascular Medicine Unit, Monash Health, Melbourne, VIC, Australia; ^3^Faculty of Health, Arts and Design, Iverson Health Innovation Research Institute, Swinburne University of Technology, Melbourne, VIC, Australia; ^4^Human Neurotransmitters Laboratory, Baker IDI Heart & Diabetes Institute, Melbourne, VIC, Australia; ^5^Clinical Obesity Research Laboratories, Baker Heart & Diabetes Institute, Melbourne, VIC, Australia

**Keywords:** polycystic ovary syndrome, sympathetic nervous system, insulin resistance, moxonidine, randomized controlled trial

## Abstract

Sympathetic nervous system (SNS) activity is increased in polycystic ovary syndrome (PCOS). Moxonidine is a centrally acting sympatholytic drug with known beneficial effects on hypertension, insulin sensitivity, dyslipidemia and inflammation. In this double-blind placebo controlled randomized clinical trial we examined the effect of moxonidine on modulating sympathetic activity and downstream metabolic abnormalities in 48 pre-menopausal women with PCOS (Rotterdam diagnostic criteria), recruited from the community (January 2013–August 2015). Participants received moxonidine (0.2 mg daily initially, up titrated to 0.4 mg daily in 2 weeks) (*n* = 23) or placebo (*n* = 25) for 12 weeks. Multiunit muscle sympathetic activity (by microneurography) and plasma noradrenaline levels were measured (primary outcomes). Fasting lipids, insulin resistance, serum androgens, and inflammatory markers were measured as secondary outcomes. Forty three women completed the trial (19 moxonidine, 24 placebo). Mean change in burst frequency (−3 ± 7 vs. −3 ± 8 per minute) and burst incidence (−3 ± 10 vs. −4 ± 12 per 100 heartbeat) did not differ significantly between moxonidine and placebo groups. Women on moxonidine had a significant reduction in hs-CRP compared to placebo group (−0.92 ± 2.3 vs. −0.04 ± 1.5) which did not persist post Bonferroni correction. There was a significant association between markers of insulin resistance at baseline and reduction in sympathetic activity with moxonidine. Moxonidine was not effective in modulating sympathetic activity in PCOS. Anti-inflammatory effects of moxonidine and a relationship between insulin resistance and sympathetic response to moxonidine are suggested which need to be further explored.

**Clinical Trial Registration Number:** (NCT01504321)

## Introduction

Polycystic ovary syndrome (PCOS) is a common endocrinopathy of reproductive age women, affecting 12–18%, with hyperinsulinemia and hyperandrogenism being the key hormonal features underpinning the pathophysiology of the disease (Dunaif, 1997; March et al., 2010; Teede et al., 2011). Additional to reproductive features, PCOS is associated with a range of metabolic abnormalities including insulin resistance (IR) and impaired glucose tolerance (IGT), gestational diabetes, type 2 diabetes, dyslipidemia, obstructive sleep apnoea (OSA) and an increased risk of cardiovascular disease (Teede et al., 2011; Shorakae et al., 2014; Hart and Doherty, 2015). This increased risk of cardiometabolic abnormalities in PCOS, at least in part, is attributed to the interrelated effects of hyperandrogenism, IR, sympathetic nervous system (SNS) dysfunction and chronic low grade inflammation (Shorakae et al., 2015).

Increasing evidence arising from animal and human studies favors the role of increased SNS activity in the pathophysiology of PCOS (Lara et al., 1993, 2002; Greiner et al., 2005; Stener-Victorin et al., 2005; Yildirir et al., 2006; Sverrisdottir et al., 2008). A recent study by our group demonstrated increased sympathetic drive, measured from multi- and single unit muscle SNS activity (MSNA), in overweight and obese women with PCOS, with the sympathoexcitation being independent of obesity and other metabolic disturbances (Lambert et al., 2015). Others have shown that modulation of SNS with electro-acupuncture, effectively lowered the concentration of ovarian nerve growth factor (NGF) (Stener-Victorin et al., 2000a) and improved insulin sensitivity and lipid profile in PCOS animal models (Mannerås et al., 2008; Johansson et al., 2010). Electro-acupuncture, physical exercise and renal denervation have effectively modulated SNS in women with PCOS and generated beneficial effects on ovulation, hyperandrogenism and oligomenorrhea (Stener-Victorin et al., 2000b, 2009; Jedel et al., 2011; Schlaich et al., 2011; Johansson et al., 2013).

Moxonidine, an imidazoline 1 agonist, acts centrally to modulate sympathetic activity and has previously been shown to reduce sympathetic activity in obese hypertensive subjects (Esler et al., 2004; Sanjuliani et al., 2006; Chazova and Schlaich, 2013) and in young overweight, but otherwise healthy, males (Lambert et al., 2017). Additional to inhibition of sympathetic drive, Imidazoline 1 agonists have been demonstrated to beneficially influence glucose and lipid metabolism and insulin sensitivity (Haenni and Lithell, 1999; Chazova et al., 2006; Fenton et al., 2006), hemodynamic and neuroendocrine parameters (Mitrovic et al., 1991), endothelial dysfunction (Topal et al., 2006) and inflammatory cytokine production (Pöyhönen-Alho et al., 2008) when used in hypertensive, overweight and insulin resistant subjects.

No data is available on moxonidine therapy and metabolic outcomes in women with PCOS, a metabolic condition underpinned by IR. Here, we aimed to study moxonidine to preferentially target the sympathetic nervous system in a randomized controlled trial in women with PCOS. We hypothesized that central sympathoinhibition with moxonidine would favorably modify sympathetic nervous activity (primary outcome) and positively influence metabolic abnormalities (secondary outcomes) in PCOS.

## Materials and methods

### Study population and design

In this double blind randomized placebo controlled trial (NCT01504321), women with PCOS were recruited through community advertisement between January 2013 and March 2015. PCOS diagnosis and eligibility was confirmed according to Rotterdam criteria, with presence of two of the following three: oligo/anovulation (or cycle length < 35 days), clinical (hirsutism) or biochemical hyperandrogenism and polycystic ovarian morphology on ultrasound (presence of 12 or more follicles measuring 2–9 mms in one or both ovaries) (Group, 2004). Hirsutism was evaluated using a modified Ferriman-Gallwey scoring (m-FG score) system (Yildiz et al., 2010) and was defined as an m-FG score above 8 in Caucasian and above 6 in Asian women.

This study protocol was approved by Ethics committees at the Alfred Hospital and Monash Health. All subjects gave written informed consent prior to participation.

### Study protocol

Overall 207 pre-menopausal women with history of PCOS were assessed for eligibility. Eligibility criteria included BMI 20–40 kg/m^2^ and age 18–45 years old. Exclusion criteria included pregnancy, diabetes, use of any medication that could interfere with sympathetic nervous system activity (i.e. SNRI antidepressants, α or β blocking anti-hypertensives) and insulin resistance within the 3 months prior to recruitment, a history of secondary hypertension (high blood pressure caused by another medical condition), cardiovascular, cerebrovascular, renal, liver, thyroid or lung disease and severe mental illness. Women who were taking oral contraceptive pills (OCP) or metformin were asked to stop the medication, use barrier contraception if desired and if no contraindications, and to go through a 3 month wash out period for OCP and 1 month washout period for metformin, prior to recruitment. PCOS diagnosis was confirmed according to the Rotterdam diagnostic criteria (Group, 2004).

**Figure 1:**
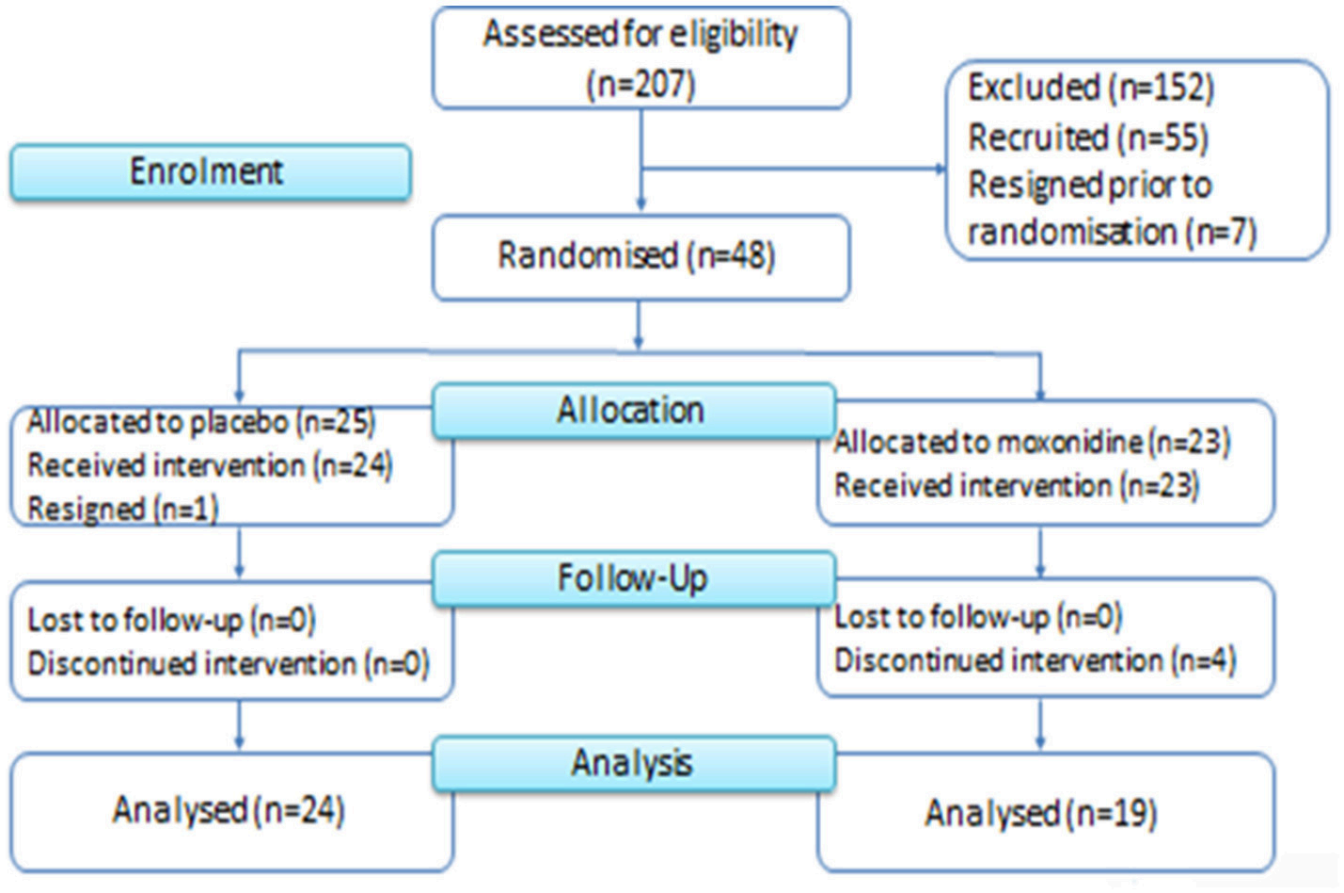
Consort diagram of recruitment and study flow.

Moxonidine and placebo capsules were prepared at the Baker Heart & Diabetes Institute using commercially available moxonidine tablets and lactose powder dispensed into identical capsules. Participants were randomized by the Alfred Hospital Clinical Trial Pharmacy and were allocated to the 12 weeks trial intervention [moxonidine (0.2 mg daily initially, up titrated to 0.4 mg daily in 2 weeks) or placebo], between June 2013 and August 2015 (Figure 1). Randomization was performed using a simple block randomization with blocks of 10 (5 moxonidine and 5 placebo in each block). All participants and investigators remained blinded until after data lockdown and analysis of results.

All participants attended the Baker Heart & Diabetes Institute (at baseline and at completion of the trial) and the Monash Center for Health Research and Implementation (at baseline, at randomization and at the three follow up visits including one after completion of the trial) for a total number of 7 visits across both sites. Clinical assessments, including all measurements, were performed by a doctor at baseline and at completion of the study. Body weight was measured in light clothes without shoes, using a digital scale. The BMI was calculated as weight (kg)/height squared (m^2^). Waist circumference was measured at the midpoint between iliac crest and the lowest rib. Total, peripheral, trunk and abdominal fat and lean mass were determined by dual energy absorptiometry using a GE, Lunar Prodigy DEXA scanner.

Muscle sympathetic nerve activity was assessed and analyzed using methods reported previously (Lambert et al., 2015). Recordings of multiunit postganglionic MSNA were made using a tungsten microelectrode (FHC, Bowdoinham, ME, USA) inserted directly into the right peroneal nerve at the fibular head. During MSNA recording, blood pressure (BP) was measured continuously using the Finometer system (Finapres Medical System BV, Amsterdam, the Netherlands), and heart rate (HR) was extracted from a 3-lead ECG. All of these parameters were digitized with a sampling frequency of 1,000 Hz (PowerLab recording system, model ML 785/8SP; ADI Instruments, Bella Vista, NSW, Australia). Resting measurements were recorded over a 15-min period and averaged. Sympathetic bursts were counted manually and expressed as burst frequency (bursts/min) and burst incidence (bursts/100 heart beats).

Fasting blood samples were drawn from participants, at baseline and at completion of the trial. Hormonal analyses were performed at Monash Pathology, Monash Health and metabolic determinations were performed at the Alfred Hospital Pathology. The Access SHBG assay was performed using a sequential two-step immunoenzymatic (“sandwich”) assay carried out on a Beckman Coulter Unicel DXI 800 (Beckman Coulter, Lane Cove, NSW, Australia). Testosterone assay was performed by high performance liquid chromatography–mass spectrometry (HPLCMS/MS) method using a liquid sample extraction (AB Sciex Triple Quad 5500 LC/MS/MS system; Mt Waverley, Vic., Australia). Free Androgen Index (FAI) was calculated as (total testosterone × 100)/SHBG. Plasma glucose and lipid profile were quantified by automated enzymatic methods (Architect C18000 analyser; Abbott Laboratories, Abbott Park, IL, USA), insulin by radioimmunoassay (Linco Research, Inc., St. Charles, MO, USA) and high-sensitivity C-reactive protein (hs-CRP) by immunoturbidimetric assay (reference range < = 5 mg/L). Insulin resistance was determined using Homeostatic Model Assessment for insulin resistance (HOMA-IR), calculated as (fasting insulin × fasting glucose/22.5). Plasma noradrenaline was determined by high performance liquid chromatography with coulometric detection as previously described (Lambert and Jonsdottir, 1998).

All medications were dispensed following randomization. Participants were reviewed every 4 weeks for collection of menstrual diary, measurement of waist and hip circumference and weight and monitoring of heart rate and blood pressure. Participants were screened for development of side effects via a questionnaire at each follow up visit. Adherence to the allocated intervention was assessed by counting the number of remaining capsules in medication containers which were collected at each follow up visit.

### Statistical analysis and sample size calculation

A sample size of 42 (21 subjects per treatment arm) was calculated a priori with 80% power to demonstrate a difference in MSNA (primary outcome) of 20% or greater (α = 0.05). This was based on data from our previous work indicating that MSNA in young overweight females is approximately 40 bursts per 100 heartbeats (standard deviation 9 bursts per 100 heartbeats) (Lambert et al., 2007, 2013).

Data were analyzed using IBM SPSS Statistics, version 22 (Armonk, NY, USA). Data are presented as mean ± SD or median (IQR) if data were skewed. Histograms were used to determine data distribution and skewed data were log transformed if appropriate. Data were analyzed for primary and secondary outcomes as per protocol. Absolute and relative change from baseline were calculated for primary and secondary outcomes. Student's *t* test was used to compare between group differences in mean absolute and relative change and paired *t* test was used for comparison of within group differences. Non-parametric methods were used for non-normally distributed variables. The robustness of analyses was confirmed by conducting Bonferroni correction several dependent and independent analysis. The corrected *p*-value (0.002) was calculated as 0.05 divided by number of tested variables (*n* = 23). Correlations with baseline characteristics were determined using Pearson's correlation coefficient. To determine associations with change in the primary outcome, regression analysis was performed including parameters with significant correlation (*p* < 0.1) and significant interaction with the study intervention (*p* < 0.1). All statistical methods were confirmed with an experienced biostatistician. Statistical significance was defined as a *p* ≤ 0.05.

## Results

Fifty-five women were recruited between January 2013 and March 2015 and PCOS diagnosis was confirmed. Seven women dropped out prior to randomization due to personal reasons. Forty-eight women were randomized and allocated to the 3 months trial intervention [moxonidine (*n* = 23) or placebo (*n* = 25)] between June 2013 and August 2015. Four women in the moxonidine group discontinued the allocated intervention; one woman became pregnant, one developed a skin rash (considered unrelated to the trial intervention) and two dropped out due to personal reasons. One woman in the placebo group withdrew from the trial prior to receiving the allocated intervention. Nineteen women in the moxonidine group and 24 women in the placebo group received the allocated intervention for 3 months and were analyzed for the primary and secondary outcomes (Figure 1).

### Baseline characteristics

The anthropometric, haemodynamic, metabolic, and hormonal characteristics of participants are presented in Table 1. Overall, there were no differences in baseline characteristics between the two groups.

**Table 1:** Baseline characteristics of participants in each group.

**Variable**	**Placebo (*n* = 24)**	**Moxonidine (*n* = 19)**
	**mean** ±***SD*** **median(IQR)**	**mean** ±***SD*** **median(IQR)**
Age (years)	31 ± 6	29 ± 6
**ANTHROPOMETRIC**		
Weight (kg)	80 ± 15	78 ± 17
BMI (kg/m^2^)	30 ± 5	29 ± 6
WC (cm)	102 ± 13	101 ± 16
WHR	0.97 ± 0.05	0.95 ± 0.06
Body fat (%)	43 ± 10	43 ± 9
**HAEMODYNAMIC**		
SBP (mmHg)	109(12)	108(9)
DBP (mmHg)	71 ± 10	69 ± 8
HR (per minute)	63 ± 8	66 ± 10
**METABOLIC**		
Fasting insulin (μU/ml)	19.4 ± 11.1	15.0 ± 4.5
Fasting glucose (mmol/L)	4.6 ± 0.6	4.6 ± 0.3
Post OGTT insulin (μU/ml)	84.9 ± 57.8	89.8 ± 52.3
Post OGTT glucose (mmol/L)	5.8 ± 1.5	5.2 ± 1.2
HOMA-IR	4.0(3.9)	3.4(2.1)
TC (mmol/L)	4.8 ± 0.8	4.8 ± 0.8
TG (mmol/L)	1.2 ± 0.7	0.9 ± 0.4
HDL (mmol/L)	1.4 ± 0.3	1.5 ± 0.4
LDL (mmol/L)	2.9 ± 0.8	3.0 ± 0.8
hs_CRP (mg/L)	2.2(3.1)	2.6(4.3)
**REPRODUCTIVE**		
Total testosterone (nmol/L)	1.5 ± 0.7	1.4 ± 0.7
SHBG (nmol/L)	39 ± 17	49 ± 19
FAI	5 ± 4.3	3.3 ± 2
FG-score	13 ± 5	14 ± 6

*Skewed data are presented as median (IQR) and log transformed data were included in the analysis. All p-values for differences between the groups were non-significant*.

*BMI, body mass index; WC, waist circumference; WHR, waist to hip ratio; SBP, systolic blood pressure; DBP, diastolic blood pressure; HR, heart rate; OGTT, oral glucose tolerance test; HOMA-IR, homeostatic model of assessment of insulin resistance; TC, total cholesterol; TG, triglycerides; HDL-C, high density lipoprotein cholesterol; LDL-C, low density lipoprotein cholesterol; hs-CRP, high sensitivity C-reactive protein; DHEAS, dehydroepiandrosterone sulfate; SHBG, sex hormone binding globulin; FAI, free androgen index*.

### Effect on sympathetic activity

Due to technical difficulties in obtaining a suitable recording site, only 31 participants (13 in the moxonidine group and 18 in the placebo group) completed MSNA assessments pre and post intervention. Mean absolute and relative changes in burst frequency, burst incidence and plasma noradrenaline levels were not different between the two groups (Table 2).

**Table 2:** Sympathetic, anthropometric, haemodynamic, metabolic, inflammatory, and reproductive parameters before and after 3 months of intervention in each group.

**Variable**	**Placebo**			**Moxonidine**			**Between groups comparison (Placebo vs. Moxonidine)**		
	**Pre**	**Post**	***p*-value^a^ within group**	**Pre**	**Post**	***p*-value^a^ within group**	**post intervention^b^**	**absolute change^c^**	**relative change^d^**
**Sympathetic activity parameter(s)**	***n*** = **18**			***n*** = **13**					
Burst frequency (per minute)	28(9)	26(9)	NS	27 ± 9	23 ± 8	NS	NS	NS	NS
Burst incidence (per 100 heartbeat)	44(12)	41(13)	NS	40 ± 12	37 ± 11	NS	NS	NS	NS
Noradrenaline (pg/ml)	254(149)	244(149)	NS	277 ± 125	291 ± 166	NS	NS	NS	NS
**Anthropometric parameter(s)**	***n*** = **24**			***n*** = **19**					
Weight (Kg)	80 ± 15	81 ± 15	NS	78 ± 17	79 ± 17	NS	NS	NS	NS
BMI (Kg/m^2^)	30 ± 5	30 ± 5	NS	29 ± 6	29 ± 6	NS	NS	NS	NS
WHR	0.97 ± 0.05	0.96 ± 0.04	NS	0.95 ± 0.06	0.96 ± 0.06	NS	NS	NS	NS
Body fat (%)	43 ± 10	42 ± 9	NS	46(9)	44(9)	NS	NS	NS	NS
**Haemodynamic parameter(s)**	***n*** = **23**			***n*** = **19**					
SBP (mmHg)	109 ± 10	110 ± 11	NS	108(9)	109(15)	NS	NS	NS	NS
DBP (mmHg)	70(10)	68(15)	NS	69 ± 8	69 ± 8	NS	NS	NS	NS
HR (per minute)	64 ± 8	62 ± 8	NS	66 ± 10	62 ± 8	NS	NS	NS	NS
**Metabolic parameter(s)**	***n*** = **22**			***n*** = **19**					
Fasting glucose (mmol/L)	4.6(0.7)	4.6(0.7)	NS	4.6 ± 0.4	4.6 ± 0.3	NS	NS	NS	NS
Post OGTT glucose (mmol/L)	5.5(2.1)	5.8(2.5)	NS	5.8 ± 1.3	5.3 ± 1.2	NS	NS	NS	NS
Fasting insulin (μU/ml)	23.2 ± 15.2	19.4 ± 11.13	NS	17 ± 7.2	14.9 ± 4.5	NS	NS	NS	NS
Post OGTT insulin (μU/ml)	74.9(99.4)	64.7(96.9)	NS	86.9 ± 53.6	89.8 ± 52.3	NS	NS	NS	NS
HOMA-IR	4(5.2)	3.2(3.2)	NS	3.5 ± 1.5	3 ± 0.9	NS	NS	NS	NS
TC (mmol/L)	4.5(1.4)	4.4(1.3)	NS	4.8 ± 0.8	4.9 ± 0.8	NS	NS	0.05	0.05
TG (mmol/L)	0.9(1.0)	0.9(0.7)	NS	0.8(0.5)	0.9(0.4)	NS	NS	0.05	0.02
HDL (mmol/L)	1.4 ± 0.3	1.4 ± 0.2	NS	1.5(0.5)	1.3(0.7)	NS	NS	NS	NS
LDL (mmol/L)	2.9 ± 0.8	2.8 ± 0.7	NS	2.9 ± 0.8	3.1 ± 0.7	NS	NS	NS	0.04
**Inflammatory parameter(s)**	***n*** = **22**			***n*** = **18**					
hs-CRP (mg/L)	2.4(3.4)	1.6(5.5)	NS	2.1(4.2)	1.4(4.3)	0.004	NS	NS	0.02
**Reproductive parameter(s)**	***n*** = **22**			***n*** = **19**					
Testosterone (nmol/L)	1.4 ± 0.7	1.3 ± 0.8	NS	1.1(0.9)	1.3(0.7)	NS	NS	NS	NS
SHBG (nmol/L)	37 ± 16	42 ± 20	0.005	49 ± 19	53 ± 20	0.03	NS	NS	NS
FAI	3.9(3.8)	3.1(3.9)	NS	2.6(2.8)	2.3(2.3)	NS	NS	NS	NS

*Skewed data are presented as median (IQR) and non-parametric methods were used for analysis*.

a *p-value for within group comparison of pre vs. post intervention data*.

b *p-value for between group (moxonidine vs. placebo) comparison of post intervention data*.

c *p-value for between group (moxonidine vs. placebo) comparison of absolute change from baseline*.

d *p-value for between group (moxonidine vs. placebo) comparison of relative change from baseline*.

*BMI, body mass index; WHR, waist to hip ratio; SBP, systolic blood pressure; DBP, diastolic blood pressure; HR, heart rate; OGTT, oral glucose tolerance test; HOMA-IR, homeostatic model of assessment of insulin resistance; TC, total cholesterol; TG, triglycerides; HDL-C, high density lipoprotein cholesterol; LDL-C, low density lipoprotein cholesterol; hs-CRP, high sensitivity C-reactive protein; DHEAS, dehydroepiandrosterone sulfate; SHBG, sex hormone binding globulin; FAI, free androgen index*.

### Effect on haemodynamic parameters

Mean absolute and relative change in SBP, DBP and HR were not different between the two groups (Table 2).

### Effect on lipid profile

The mean absolute (−0.1 ± 0.3 vs. 0.2 ± 0.6, *p* = 0.05 for TC and −0.1 ± 0.4 vs. 0.1 ± 0.3, *p* = 0.05 for TG respectively) and relative change (−2 ± 7% vs. 4 ± 12%, *p* = 0.05 for TC, −4 ± 30% vs. 19 ± 33%, *p* = 0.02 for TG and −2 ± 10% vs. 8 ± 18%, *p* = 0.04 for LDL-C respectively) were different between the placebo and moxonidine groups (Table 2). These became non-significant post Bonferroni correction for number of analysis (*p* > 0.002).

### Effect on inflammatory markers

There was a reduction in hs-CRP within the moxonidine group [2.1(4.2) vs. 1.4(4.3), *p* = 0.004] and the mean relative change in hs-CRP was different between the placebo and moxonidine groups (Table 2). These became non-significant post Bonferroni correction for number of analysis (*p* > 0.002).

### Effect on reproductive hormone profile

SHBG increased within both moxonidine and placebo groups (49 ± 19 vs. 53 ± 20, *p* = 0.03 and 37 ± 16 vs. 42 ± 20, *p* = 0.005 respectively) which became non-significant post Bonferroni correction for number of analysis (*p* > 0.002) (Table 2). The mean absolute and relative change were not different between the two groups.

### Associations with change in sympathetic function

**Figure 2:**
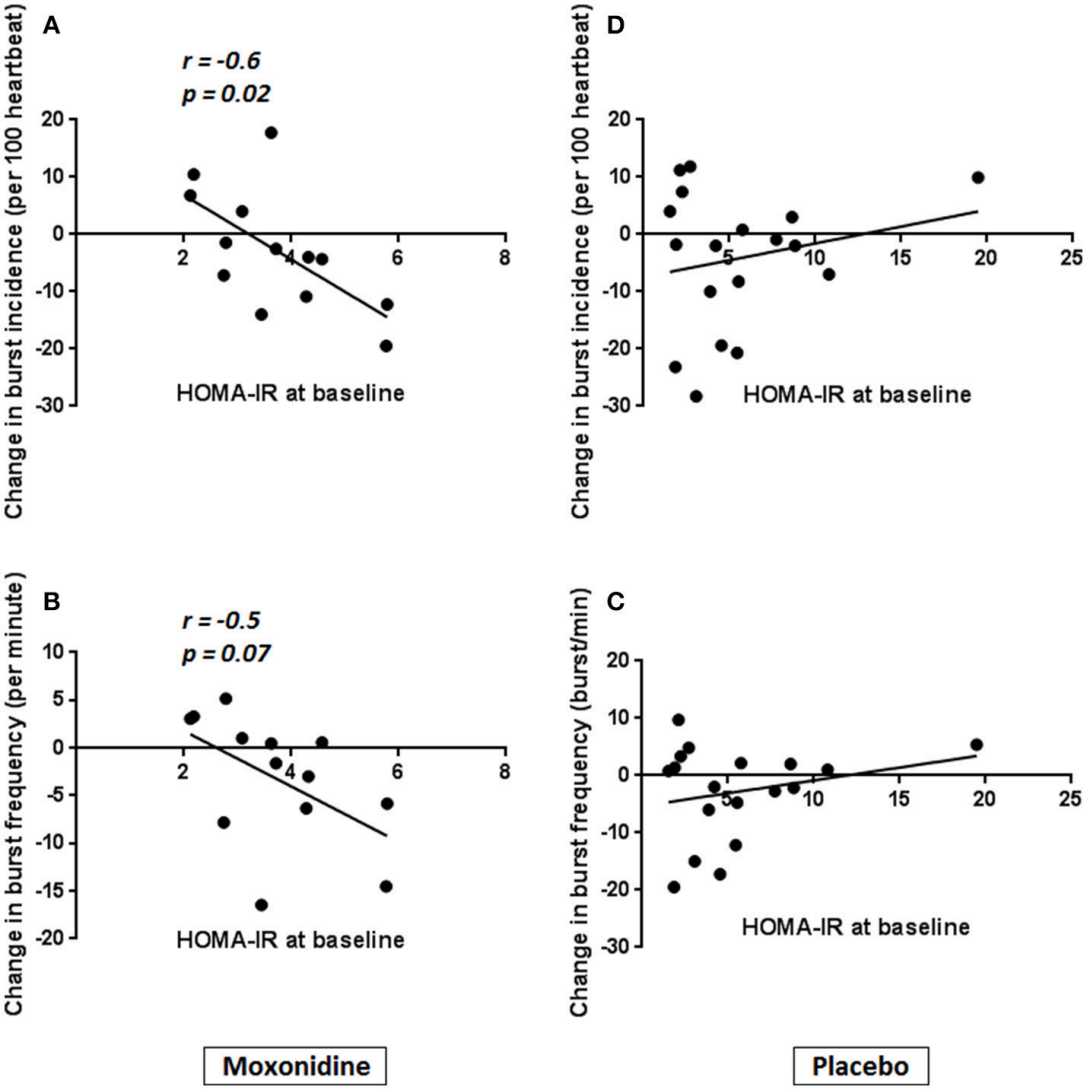
Correlation of HOMA-IR with magnitude of change in burst incidence and burst frequency in Moxonidine vs. placebo group. In the moxonidine group, reduction in burst incidence **(A)** and burst frequency **(B)** correlated with HOMA-IR at baseline. There was no correlation of change in MSNA with baseline HOMA-IR in the placebo group **(C,D)**.

In the moxonidine group, reduction in burst frequency correlated with fasting insulin, post OGTT insulin and post OGTT glucose at baseline. Reduction in burst incidence also correlated with fasting insulin and HOMA-IR at baseline (Table 3, Figure 2). There was no correlation of change in MSNA with markers of insulin resistance in the placebo group. We tested for interaction between the markers of insulin resistance and intervention in sympathetic response to moxonidine. The interaction was significant for post OGTT glucose (*p* = 0.04) and approached significance for fasting insulin (*p* = 0.06) when tested for mean change in burst frequency. It was significant for fasting insulin (*p* = 0.03) and HOMA-IR (*p* = 0.03) when tested for mean change in burst incidence.

**Table 3:** Correlation of baseline insulin sensitivity with magnitude of change in sympathetic function in moxonidine group.

**Mean change MSNA**			**Post OGTT glucose**	**Fasting insulin**	**Post OGTT insulin**	**HOMA-IR**
Mean change in Burst frequency	Correlation analysis	*R*	−0.608	−0.546	−0.633	−0.522
		*P*-value	0.03	0.05	0.02	0.07
	Interaction with the study intervention	*P*-value	0.04	0.06	0.09	0.08
	Univariate regression analysis	*R*^2^	0.4	0.3	0.4	0.3
		B coefficient	−3.1	−0.6	−0.1	−2.9
		*P*-value	0.03	0.05	0.02	0.07
Mean change in Burst incidence	Correlation analysis	*R*	−0.451	−0.648	−0.433	−0.628
		*P*-value	0.1	0.017	0.1	0.02
	Interaction with the study intervention	*P*-value	0.09	0.03	0.2	0.03
	Univariate regression analysis	*R*^2^	0.2	0.4	0.2	0.4
		B coefficient	−3.6	−1.1	−0.1	−5.6
		*P*-value	0.1	0.02	0.1	0.02

*OGTT, oral glucose tolerance test; HOMA-IR, homeostatic model of assessment of insulin resistance*.

On univariate regression analysis in the moxonidine group mean change in burst frequency was significantly associated with post OGTT glucose and fasting insulin and mean change in burst incidence was significantly associated with fasting insulin and HOMA-IR at baseline. All *p*-values were non-significant (*p* > 0.1) for the placebo group.

### Adherence to allocated intervention and adverse effects

Ten participants (6 in the placebo group and 4 in the moxonidine group) failed to return the empty medication containers for assessment of adherence to intervention. In the remaining participants, adherence to the allocated intervention was 97% in the moxonidine group and 100% in the placebo group. No serious adverse events were reported. The most commonly reported side effects across all visits included lack of energy (54%), dry mouth (52%), headache (44%), drowsiness (38%), and sleep difficulties (34%) in the moxonidine group and lack of energy (64%), headache (55%), dry mouth (48%), drowsiness (43%), and sleep difficulties (41%) in the placebo group respectively. The frequency of reported side effects did not change significantly as the trial progressed (Supplementary Table 1).

## Discussion

In this study, we investigated the effect of moxonidine on modulating sympathetic activity and downstream metabolic abnormalities in a 12 weeks double blind randomized placebo controlled trial of moxonidine vs. placebo in 48 pre-menopausal women with PCOS. We found that moxonidine did not reduce sympathetic activity when compared to placebo in women with PCOS. Women on moxonidine had a reduction in hs-CRP compared to the placebo group which became non-significant post Bonferroni correction. Total cholesterol, LDL and triglyceride levels slightly increased in the moxonidine group and decreased in the placebo group which did not reach statistical significance. We found a significant interaction between markers of insulin resistance and reduction in sympathetic activity with moxonidine.

Ovarian sympathetic activity is most likely increased in polycystic ovaries. Human studies have demonstrated a higher density of catecholaminergic fibers in polycystic ovaries (Heider et al., 2001). Moreover, human (Dissen et al., 2009) and animal (Lara et al., 2000) studies have demonstrated an association between increased intra-ovarian synthesis of nerve growth factor (NGF) and polycystic ovaries. While increased ovarian sympathetic activity results in increased intra-ovarian androgen synthesis in response to gonadotrophins (Barria et al., 1993), some evidence suggests the increased ovarian sympathetic outflow may even precede development of arrested follicles in polycystic ovaries (Lara et al., 1993). In an experimental model sympathetic denervation of the ovary prevented the development of ovarian cysts (Barria et al., 1993). Previous interventions using low frequency electro-acupuncture and renal denervation modulated sympathetic over activity in women with PCOS with reduction in MSNA and noradrenaline spillover to plasma (Stener-Victorin et al., 2009; Jedel et al., 2011; Schlaich et al., 2011). Considering both of these procedures offer discrete and organ specific alterations in sympathetic tone, including at the level of ovaries with electroacupuncture (Stener-Victorin et al., 2003, 2004, 2006), it is plausible that the increased sympathetic tone previously documented in women with PCOS (Sverrisdottir et al., 2008; Lambert et al., 2015) also included activation of the sympathetic drive to the ovaries.

Moxonidine effectively reduced MSNA and noradrenaline levels, in comparison to placebo, when trialed in insulin resistant, overweight/obese or hypertensive participants (Greenwood et al., 2000; Sanjuliani et al., 2006; Dorresteijn et al., 2013) with variable metabolic and haemodynamic effects (Haenni and Lithell, 1999; Chazova et al., 2006; Sanjuliani et al., 2006; Topal et al., 2006; Dorresteijn et al., 2013; Lambert et al., 2017). When trialed in young, overweight men, moxonidine 0.4 mg/day for 24 weeks resulted in reduction in MSNA and substantially decreased the clinic systolic BP but failed to improve metabolic parameters (Lambert et al., 2017). The contributors to the lack of effect of moxonidine on MSNA in the present study are unclear. With 72% of participants completed MSNA assessments pre and post intervention, we were 86% powered to detect a 25% reduction in sympathetic activity in this study. Given the high level of adherence to study medication in both groups, the lack of effect of moxonidine on MSNA suggests either we were underpowered to detect a smaller reduction of < 25% in SNS activity by moxonidine, or that the sympathetic drive in women with PCOS, at least in part, has its origins in brain regions other than those influenced by imidazoline agonists; with further research needed in future. Moreover MSNA is affected by sex and age (Ng et al., 1993) and is influenced by visceral adiposity (Alvarez et al., 2002) and phase of the ovarian cycle (Carter et al., 2013). The potential role of these factors influencing the physiological response to moxonidine in women with PCOS also remains unknown and deserves further attention.

There is a close inter-relation between insulin sensitivity and sympathetic activity, with insulin resistance and hyperinsulinemia being associated with SNS over activity (Kaaja and Poyhonen-Alho, 2006; Dampney, 2011). In our study, the magnitude of change in MSNA correlated significantly with baseline insulin resistance in the moxonidine group. We demonstrated that mean change in burst frequency significantly correlated with post OGTT glucose and fasting insulin and mean change in burst incidence significantly correlated with fasting insulin and HOMA-IR at baseline. This is consistent with previous studies demonstrating the effectiveness of moxonidine in improving sympathetic and haemodynamic profiles in insulin resistant populations (Sanjuliani et al., 2006; Dorresteijn et al., 2013).

It is reported previously that total cholesterol and triglyceride levels significantly reduced (Lumb et al., 2004; Ebinç et al., 2008) or remained unchanged (Elisaf et al., 1999) with moxonidine in overweight and obese hypertensive participants. In this study a small, non-clinically significant deterioration in fasting lipids is shown in the moxonidine group. Clearly the impact of moxonidine on fasting lipid profile remains unclear. Moxonidine has reduced circulating TNF-α and IL-6 levels in spontaneously hypertensive rat models suggestive of a potential direct effect on cytokine signaling and inflammation (Aceros et al., 2011). Moxonidine has also resulted in reduction in TNF-α levels in 87 overweight postmenopausal women with hypertension (Pöyhönen-Alho et al., 2008). In our study, women on moxonidine had reduction in hs-CRP levels which became non-significant post Bonferroni correction. While this is potentially is explained by our small sample size and lack of power for detection of a change in this secondary outcome, the clinical relevance of such finding and a potential modulatory effect of moxonidine on inflammation needs to be explored further in future studies.

### Strength and limitations

To the best of our knowledge, our study is the first double blind randomized clinical trial of pharmacological sympathetic modulation, in this case with moxonidine vs. placebo, in women with PCOS. The women were community recruited and we followed a robust methodology including using the gold standard microneurography for direct measurement of sympathetic activity. Although microneurography is a powerful technique, obtaining an appropriate recording site, particularly in overweight subjects, can be challenging for both the investigator and participant. Further limitations include the use of indirect measures of insulin resistance and that participant recruitment and maintenance throughout the study was difficult given the large commitment required.

## Conclusion

Overall moxonidine was not effective in modulating sympathetic activity in women with PCOS based on this study. We found a significant relationship between insulin resistance and sympathetic response to moxonidine, suggesting large clinical trials are needed to evaluate the effectiveness of this medication potentially targeting the most insulin resistant PCOS cases. We also report an anti-inflammatory effect of moxonidine in women with PCOS which needs further exploration in terms of the underlying mechanistic pathways.

## Author contributions

SS contributed to recruitment, data collection and data management, statistical analysis and data interpretation and manuscript preparation. HT, GL, JD, and EJ were involved in study design. HT also contributed to statistical analysis, data interpretation and critical revision of the manuscript. GL also contributed to data interpretation and critical revision of the manuscript. JD also contributed to critical revision of the manuscript. EJ also contributed to recruitment, data collection and critical revision of the manuscript. EL participated in muscle sympathetic nerve measurements, data collection and critical revision of the manuscript. CI participated in recruitment, data collection and data management and critical revision of the manuscript. BdC was involved in statistical analysis and critical revision of the manuscript.

### Conflict of interest statement

The laboratory of GL has recently received research funding from Medtronic, Servier Australia, Abbott Pharmaceuticals, Allergan Inc. GL has acted as a consultant for Medtronic and has received honoraria from Medtronic, Pfizer and Wyeth Pharmaceuticals for presentations. These organizations played no role in the design, analysis or interpretation of data described here, nor in the preparation, review, or approval of the manuscript. The remaining authors declare that the research was conducted in the absence of any commercial or financial relationships that could be construed as a potential conflict of interest.
