# Supplementary material for: Effect of Central Sympathoinhibition With Moxonidine on Sympathetic Nervous Activity in Polycystic Ovary Syndrome—A Randomized Controlled Trial
Source: Front Physiol. 2018 Oct 25;9:1486. doi: 10.3389/fphys.2018.01486 (PMC6210452; doi:10.3389/fphys.2018.01486)
Supplement: Supplementary file 1 [file Table_1.docx]

**Table S1.** Reported adverse effects

| **Reported adverse effect** | **Placebo** | | | | **Moxonidine** | | | |
| --- | --- | --- | --- | --- | --- | --- | --- | --- |
|  | **Visit 3** | **Visit 4** | **Visit 5** | **Visit 6** | **Visit 3** | **Visit 4** | **Visit 5** | **Visit 6** |
| **Dry mouth** | Mil (5)  Mod (2)  Sev (1) | Mil (3)  Mod (4)  Sev (1) | Mil (6)  Mod (4)  Sev (1) | Mil (5)  Mod (4)  Sev (3) | Mil (3)  Mod (4)  Sev (0) | Mil (7)  Mod (4)  Sev (0) | Mil (10)  Mod (4)  Sev (0) | Mil (5)  Mod (6)  Sev (0) |
| **Headache** | Mil (11)  Mod (4)  Sev (1) | Mil (10)  Mod (5)  Sev (1) | Mil (8)  Mod (4)  Sev (1) | Mil (4)  Mod (7)  Sev (1) | Mil (6)  Mod (2)  Sev (1) | Mil (8)  Mod (2)  Sev (0) | Mil (8)  Mod (2)  Sev (0) | Mil (10)  Mod (0)  Sev (0) |
| **Lack of energy** | Mil (7)  Mod (6)  Sev (3) | Mil (11)  Mod (5)  Sev (3) | Mil (9)  Mod (4)  Sev (2) | Mil (10)  Mod (2)  Sev (2) | Mil (8)  Mod (5)  Sev (0) | Mil (5)  Mod (6)  Sev (0) | Mil (8)  Mod (5)  Sev (0) | Mil (7)  Mod (12)  Sev (1) |
| **Dizziness** | Mil (5)  Mod (1)  Sev (0) | Mil (5)  Mod (2)  Sev (2) | Mil (3)  Mod (3)  Sev (0) | Mil (5)  Mod (1)  Sev (2) | Mil (6)  Mod (0)  Sev (0) | Mil (4)  Mod (2)  Sev (0) | Mil (7)  Mod (2)  Sev (1) | Mil (4)  Mod (0)  Sev (0) |
| **Drowsiness** | Mil (7)  Mod (4)  Sev (1) | Mil (4)  Mod (6)  Sev (1) | Mil (7)  Mod (2)  Sev (2) | Mil (8)  Mod (0)  Sev (2) | Mil (7)  Mod (2)  Sev (0) | Mil (5)  Mod (5)  Sev (0) | Mil (6)  Mod (2)  Sev (1) | Mil (6)  Mod (1)  Sev (0) |
| **Nausea** | Mil (6)  Mod (1)  Sev (0) | Mil (5)  Mod (4)  Sev (2) | Mil (7)  Mod (2)  Sev (0) | Mil (4)  Mod (1)  Sev (1) | Mil (3)  Mod (0)  Sev (0) | Mil (1)  Mod (0)  Sev (0) | Mil (3)  Mod (0)  Sev (1) | Mil (3)  Mod (1)  Sev (0) |
| **Sleeping difficulty** | Mil (5)  Mod (6)  Sev (1) | Mil (7)  Mod (2)  Sev (2) | Mil (4)  Mod (4)  Sev (1) | Mil (9)  Mod (2)  Sev (0) | Mil (4)  Mod (3)  Sev (0) | Mil (4)  Mod (0)  Sev (2) | Mil (7)  Mod (2)  Sev (0) | Mil (4)  Mod (4)  Sev (1) |
| **Flushed skin** | Mil (3)  Mod (1)  Sev (0) | Mil (2)  Mod (0)  Sev (0) | Mil (1)  Mod (2)  Sev (0) | Mil (2)  Mod (0)  Sev (1) | Mil (6)  Mod (1)  Sev (0) | Mil (4)  Mod (1)  Sev (0) | Mil (2)  Mod (2)  Sev (0) | Mil (2)  Mod (1)  Sev (0) |
| **Limb swelling** | Mil (4)  Mod (0)  Sev (0) | Mil (2)  Mod (0)  Sev (0) | Mil (3)  Mod (0)  Sev (0) | Mil (1)  Mod (1)  Sev (0) | Mil (1)  Mod (0)  Sev (0) | Mil (1)  Mod (0)  Sev (0) | Mil (2)  Mod (0)  Sev (0) | Mil (2)  Mod (0)  Sev (0) |
| **Facial swelling** | Mil (4)  Mod (0)  Sev (0) | Mil (1)  Mod (0)  Sev (1) | Mil (4)  Mod (0)  Sev (0) | Mil (1)  Mod (1)  Sev (0) | Mil (0)  Mod (0)  Sev (0) | Mil (1)  Mod (0)  Sev (0) | Mil (0)  Mod (0)  Sev (0) | Mil (2)  Mod (0)  Sev (0) |
| **Shortness of breath or swallowing difficulty** | Mil (2)  Mod (0)  Sev (0) | Mil (2)  Mod (0)  Sev (1) | Mil (2)  Mod (1)  Sev (0) | Mil (3)  Mod (0)  Sev (0) | Mil (4)  Mod (1)  Sev (0) | Mil (3)  Mod (1)  Sev (0) | Mil (3)  Mod (0)  Sev (1) | Mil (1)  Mod (0)  Sev (1) |
| Note: Adverse effects at each visit are reported as incidence (number of cases) according to the reported severity: Mil, mild; Mod, moderate; Sev, severe. Visit 3, at randomisation; V4, 4 weeks post intervention; Visit 5, 8 weeks post intervention; Visit 6, 12 weeks post intervention | | | | | | | | |
